# Changes in heart rate variability and hemodynamics of adolescents within the frontal cortex in response to face emotional stimulation

**DOI:** 10.1371/journal.pone.0326204

**Published:** 2025-07-07

**Authors:** Sung Ah Chung, Hyunchan Hwang, Hee Jin Kim, Ji Sun Hong, Sun Mi Kim, Doug Hyun Han

**Affiliations:** 1 Department of Psychiatry Chung Ang University Hospital, Seoul, Republic of Korea,; 2 Department of Psychiatry Chung Ang University Gwangmyeong Hospital, Gwangmyeong, Republic of Korea; Hangzhou Normal University, CHINA

## Abstract

**Background:**

Functional near-infrared spectroscopy (fNIRS) and heart rate variability (HRV) are commonly utilized biomarkers for assessing emotional states. This study hypothesizes that emotional perception—particularly the experience and variability of unpleasant emotions in adolescents—may be characterized by reduced HRV and increased or dysregulated frontal lobe activity, indicative of impaired emotional and autonomic regulation.

**Materials and Methods:**

A total of 55 adolescents were enrolled in this study. After completing clinical questionnaires, resting-state HRV and fNIRS data were collected from all participants over a 200-second period. Following a 10-second intermission, HRV and fNIRS were simultaneously recorded during a 192-second positive emotional perception task. After a subsequent 30-second rest, the same procedures were repeated during a negative emotional perception task.

**Results:**

A higher correction rate of unpleasant facial emotional perception—defined as the proportion of emotional stimuli (positive, negative, and neutral expressions) interpreted as unpleasant—was significantly associated with reduced HRV, as evidenced by lower high-frequency (HF) power and decreased standard deviation of normal-to-normal intervals (SDNN). Moreover, this correction rate positively correlated with the differential accumulation of oxygenated hemoglobin (ΔaccHbO₂) in the left dorsolateral prefrontal cortex (DLPFC), suggesting increased cortical engagement during the processing of negatively perceived stimuli. In contrast, the correction rate of pleasant facial emotional perception showed a negative correlation with ΔaccHbO₂ in the same region. Additionally, both unpleasant-SDNN and unpleasant-HF values were negatively correlated with ΔaccHbO₂ in the left DLPFC.

**Conclusions:**

In adolescents, the perception of negative emotions is associated with individual differences in depression and anxiety levels. Furthermore, the perception of negative emotions demonstrates significant associations with alterations in HRV and neural activity within the left DLPFC. These findings also support a potential relationship between autonomic function and frontal lobe activation during the processing of unpleasant emotional stimuli.

## 1. Introduction

### 1.1. Emotional perception and regulation in adolescents

Adolescence is a critical developmental period that bridges childhood and adulthood, characterized by substantial physical, psychological, and emotional changes. This stage is often marked by emotional instability and heightened mood variability [1]. During this period, adolescents experience a wide spectrum of emotions, encompassing both positive and negative valences. However, due to ongoing neurodevelopmental maturation, they may have difficulty effectively interpreting and expressing these emotional states. Emotional perception involves the ability to detect and interpret social cues—including facial expressions, body language, and vocal intonation—during interpersonal interactions [2]. In contrast, emotion regulation refers to the processes by which individuals initiate, suppress, or modify emotional experiences, thoughts, physiological responses, and behaviors [2].

Adolescents with mood disorders such as depression frequently present with symptoms including social withdrawal, irritability (manifesting as grumpiness, hostility, low frustration tolerance, and outbursts of anger), and an increased sensitivity to perceived rejection [3]. At this developmental juncture, emotional responses are still forming, and adolescents may experiment with different ways of expressing their emotions [1]. This immaturity in emotional perception and regulation may contribute to the emergence of various psychiatric issues, including oppositional behaviors, school refusal, depression, and substance use disorders [4]. Therefore, the timely and accurate assessment of emotional responses and mood fluctuations in adolescents is essential for promoting favorable psychosocial outcomes, including adaptive social behavior, healthy cognitive and motivational processes, emotional well-being, and a positive outlook on the future [5,6].

Currently, standardized self-report instruments such as the Beck Depression Inventory, Montgomery–Åsberg Depression Rating Scale, and Mood Disorder Questionnaires are widely employed to assess adolescents’ emotional states. However, these tools are inherently subjective and may be limited in their ability to capture nuanced or fluctuating emotional dynamics [7].

Consequently, there is a growing demand for alternative assessment methods capable of offering objective and differentiated insights into adolescent emotional functioning.

### 1.2. Heart rate variability and brain activity changes in response to emotional stimulation

Heart rate variability (HRV) is widely recognized as a biomarker of parasympathetic nervous system activity [6–7]. Sustained reductions in HRV are indicative of impaired regulation across physiological, emotional, cognitive, and behavioral domains, and are associated with increased risk for adverse health outcomes and poorer self-perceived health status [8]. Common time-domain measures of HRV include the standard deviation of all inter-beat intervals (SDNN), the root mean square of successive differences between normal heartbeats (RMSSD), and the relative power of the high-frequency (HF) band. Notably, RMSSD, pNN50, and HF are considered robust indices of parasympathetic nervous system (PNS) function [9]. While SDNN reflects combined sympathetic and parasympathetic influences, in short-term resting conditions, parasympathetic modulation predominantly governs HRV fluctuations [9]. These indices are extensively utilized in psychophysiological research to assess autonomic flexibility in relation to self-regulation and physical health outcomes [10].

HRV is associated with a range of physical and psychological health conditions, including cardiovascular disease, insomnia, chronic pain, and all-cause mortality [11]. In the context of mental health, HRV has been linked to common psychiatric disorders such as depression and anxiety [12]. Empirical studies have demonstrated that HRV is sensitive to emotional arousal and mood variability. For example, Kemp et al. reported that depressive states are characterized by reduced vagal tone, as indexed by HRV, with lower HRV correlating inversely with depression severity [13]. In particular, HRV exhibits significant associations with negative affective states, including depressive and anxious symptoms. Adolescents with maladaptive emotional regulation styles tend to exhibit elevated and more erratic heart rate patterns [14].

In addition to peripheral autonomic markers, changes in brain activation patterns have been observed in response to emotional fluctuations. Functional MRI (fMRI) studies have shown that neural activity is particularly responsive to negative emotional expressions [15]. For instance, Schneider et al. (2000) identified heightened activation in the left amygdala during mood states characterized by sadness and negative affect [16]. Notably, activation within the prefrontal cortex (PFC) is closely linked to emotional processing, particularly of negative stimuli [17]. The lateral PFC plays an essential role in higher-order cognitive functions and the self-regulation of emotion. Lesion studies have implicated the dorsolateral prefrontal cortex (DLPFC) in social cognition, including the interpretation of emotional cues [18,19]. Additional research suggests that regions within the PFC, such as the DLPFC and frontopolar cortex, are engaged during emotion regulation and cognitive control tasks, with increased activation observed in response to unpleasant emotional stimuli [20].

Among neuroimaging tools, functional magnetic resonance imaging (fMRI) and functional near-infrared spectroscopy (fNIRS) have emerged as key methods for identifying neural correlates of emotional disturbances. fNIRS is a non-invasive optical technique that uses specific wavelengths of light to measure temporal changes in oxygenated (HbO) and deoxygenated (HbR) hemoglobin concentrations [21]. The forehead region, including the frontopolar and anterior lateral PFC, is particularly well-suited for fNIRS assessments. Compared to other imaging modalities, fNIRS offers advantages such as comfort, minimal setup, high spatial and temporal resolution, and suitability for pediatric and adolescent populations due to the absence of radiation exposure. Furthermore, fNIRS is relatively cost-effective and allows for the direct assessment of neural activation as well as the associated hemodynamic and metabolic responses [22]. Accordingly, biomarker measures such as HRV, in conjunction with neuroimaging modalities like fNIRS, hold considerable promise for advancing the objective assessment of emotional reactivity and perception.

### 1.3. Hypothesis

We hypothesize that emotional perception, particularly the recognition and processing of unpleasant emotions during adolescence, is associated with alterations in heart rate variability and activity within the frontal lobe.

## 2. Methods

### 2.1. Participant recruitment

A total of 55 adolescents were recruited for this study through flyer advertisements between January 2, 2023, and December 1, 2023. The inclusion criteria were: (1) age between 13 and 18 years, and (2) absence of any diagnosed psychiatric disorders or medical illnesses. Exclusion criteria included: (1) history of head trauma with loss of consciousness, seizure disorders, multiple sclerosis, brain tumors, or cerebrovascular accidents; (2) history of substance abuse; and (3) intelligence quotient (IQ) below 80. The study protocol was approved by the Institutional Review Board of Chung Ang University Hospital (approval number: 2210-009-526). Written informed consent was obtained from all participants.

### 2.2 Procedure of research protocol

**Fig 1:**
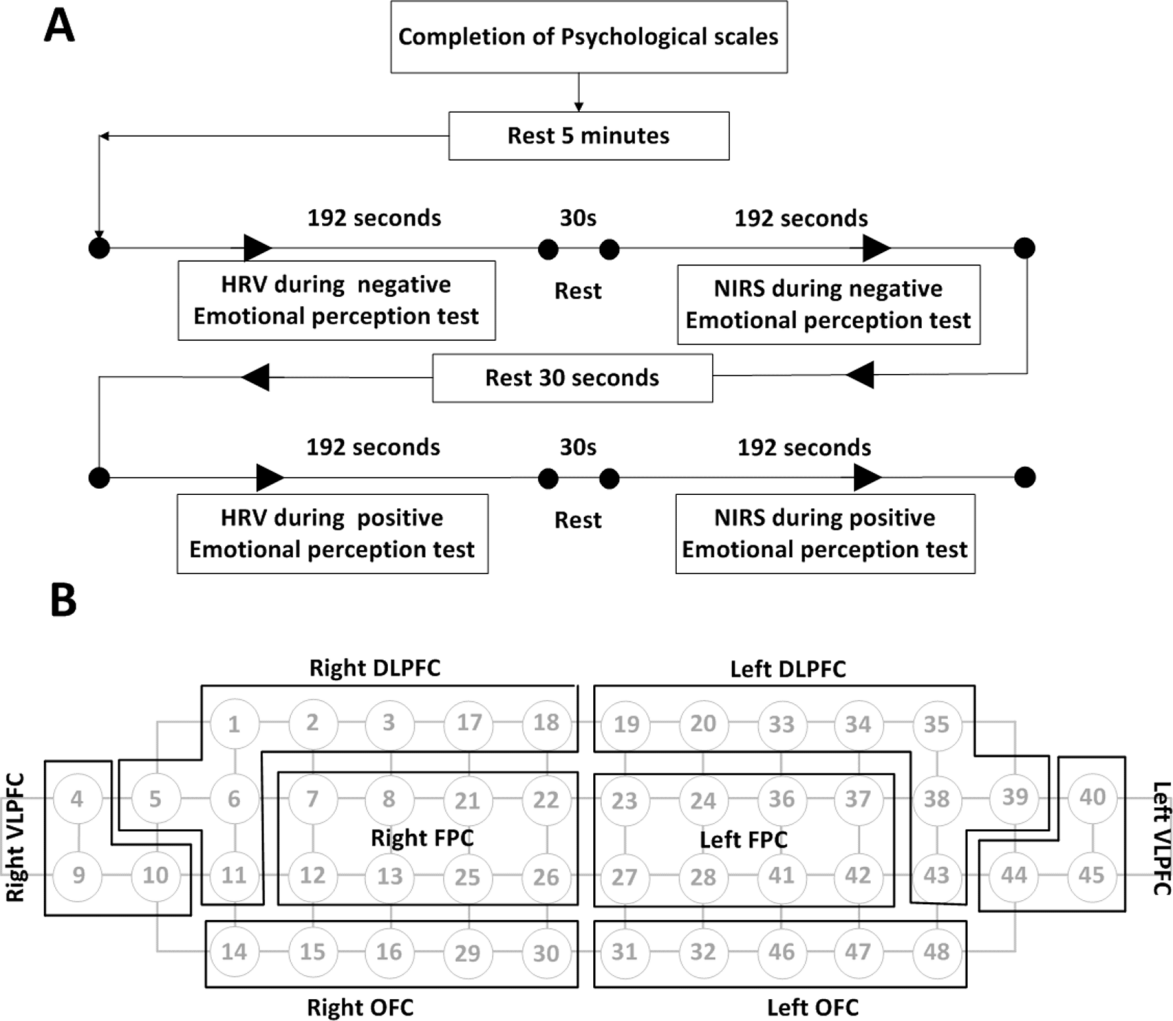
Procedure of research and NIRS. NIRS: near-infrared scopre, HRV: heart rate variability, DLPFC: dorsolateral prefrontal cortex, FPC: frontopolar cortex, OFC: orbitofrontal cortex.

Following the completion of clinical questionnaires, resting-state HRV and fNIRS data were recorded for 200 seconds. After a 10-second break, HRV and fNIRS measurements were simultaneously obtained during a 192-second task involving positive emotional perception. A 30-second rest period followed, after which the same procedures were repeated during a negative emotional perception task (Fig 1A).

### 2.3. Assessment of psychological symptoms

Depressive symptoms were assessed using the Beck Depression Inventory-II (BDI-II) [24], a 21-item self-report instrument rated on a 4-point Likert scale (0–3), yielding a total score ranging from 0 to 63. Anxiety was assessed using the Beck Anxiety Inventory (BAI) [25], which also consists of 21 self-report items rated on the same 4-point scale. Both the BDI-II and BAI demonstrated high internal consistency in this sample, with Cronbach’s alpha coefficients of 0.89 and 0.95, respectively [24,25].

### 2.4. Assessment of hemodynamic changes in the prefrontal cortex

Prefrontal cortex hemodynamic activity was assessed using the NIRSIT system, which employs 24 laser diodes (emitting at 780 nm and 850 nm) and 32 photodetectors, operating at a sampling frequency of 8.138 Hz. Although sources and detectors were positioned 1.5 cm apart, only channels with 3 cm spacing were analyzed. A total of 48 channels were established. Signals at each wavelength were processed using a band-pass filter (0.00–0.1 Hz) to minimize noise from ambient light and physiological motion. Channels with a signal-to-noise ratio below 30 dB were excluded from the analysis to ensure data integrity. Hemodynamic responses during facial emotional stimulation were calculated using the modified Beer–Lambert law [27]. For each participant, multiple trial data were block-averaged and subsequently aggregated into a grand average. The accumulated oxygenated hemoglobin (accHbO₂) values derived from these recordings were used to estimate cortical activation.

The mean and standard deviation of accHbO₂ were computed for regions of interest (ROIs) within the dorsolateral prefrontal cortex (DLPFC), frontopolar cortex (FPC), and orbitofrontal cortex (OFC) bilaterally, corresponding to Brodmann area 46. Specifically, the right DLPFC included channels 1, 2, 3, 5, 6, 11, 17, and 18, while the left DLPFC included channels 19, 20, 33, 34, 35, 38, 39, and 43. The right and left FPC included channels 7, 8, 12, 13, 21, 22, 25, and 26, and channels 23, 24, 27, 28, 36, 37, 41, and 42, respectively. The right OFC encompassed channels 14, 15, 16, 29, and 30, while the left OFC encompassed channels 31, 32, 46, 47, and 48 (Fig 1B).

### 2.5. Procedure for facial emotional stimulation

**Fig 2:**
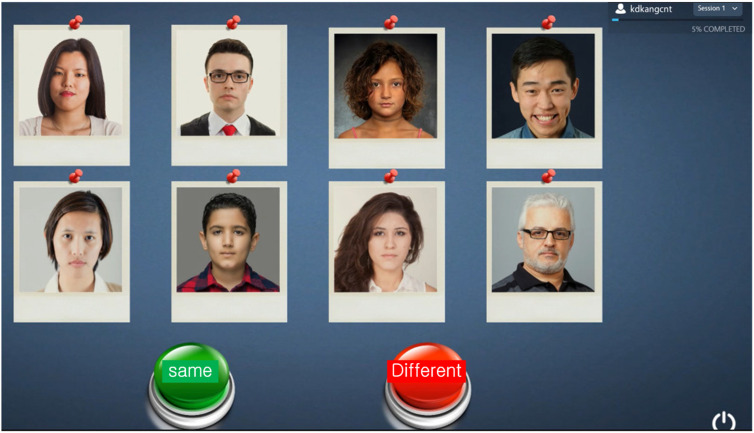
Emotional stimulation test.

A total of 66 emotional face stimuli (22 pleasant, 22 unpleasant, and 22 neutral) were extracted from the CNT emotional perception test, which had been validated in a prior study [23]. The original test comprised 128 questions, each displaying 2–8 facial images on a 10.0-inch tablet device (Galaxy SM-X810, Samsung, Korea). Participants were instructed to press either the “Same” or “Different” button based on their perception of the emotional content of the faces. Each stimulus was presented for 3 seconds (Fig 2).

For this study, a modified version of the CNT test was implemented to administer facial emotional stimulation. In the pleasant condition, stimuli included (1) all pleasant faces (AP), (2) all pleasant faces with one neutral face excluded (APeN), (3) all neutral faces (AN), and (4) all neutral faces with one pleasant face excluded (ANeP). These face groups were presented in sets of two, four, six, or eight. The four stimulus combinations and the four set sizes were randomized across four repetitions (4 combinations × 4 repetitions × 4 set sizes), resulting in 64 pleasant-face stimulations over a total duration of 192 seconds. The unpleasant condition followed a parallel structure, consisting of (1) all unpleasant faces (AUP), (2) all unpleasant faces with one neutral face excluded (AUPeN), (3) all neutral faces (AN), and (4) all neutral faces with one unpleasant face excluded (ANeUP). These were also presented in sets of two to eight faces, yielding 64 unpleasant-face stimulations over 192 seconds.

### 2.6. Assessment of heart rate variability

Based on previous research investigating HRV in adolescents with mood disorders [13,26], five key HRV parameters were selected to minimize the risk of multiple comparisons: high-frequency power (HF), low-frequency power (LF), LF/HF ratio, root mean square of successive differences (RMSSD), and standard deviation of all normal-to-normal intervals (SDNN).

HRV data were acquired using a finger photoplethysmography sensor (BioSense Creative Co., Ltd., Seoul, Korea) incorporating a pulse oximeter. Data collection was performed via a tablet personal computer equipped with a biomarker monitoring application. Both time-domain and frequency-domain analyses were conducted to derive HRV indices. Data were sampled at 30 Hz, with measurements recorded approximately every two seconds. For frequency-domain analysis, a Fast Fourier Transform (FFT) was applied to decompose the pulse wave signal into its LF and HF components. Time-domain parameters such as pNN20, pNN50, SDNN, and RMSSD were calculated using a sliding window approach, with a window size of 30 inter-beat interval (IBI) data points. Each time a new IBI was recorded, the indices were recalculated.

### 2.7. Statistical analysis

The normality of the distributions for clinical scale scores (BDI and BAI), HRV parameters, and accumulated oxygenated hemoglobin (accHbO₂) values was assessed using the Shapiro–Wilk test. Changes in accHbO₂ (ΔaccHbO₂) were calculated by subtracting resting-state NIRS values from those obtained during facial emotional stimulation. Similarly, changes in HRV indices were computed by subtracting baseline HRV values from those recorded during facial stimulation.

Pearson correlation analyses were conducted to examine the relationships between clinical scale scores and emotional perception correction rates, as well as between correction rates for unpleasant emotional perception and HRV parameters. The same statistical approach was used to evaluate associations between facial stimulation test scores and ΔaccHbO₂ values in the dorsolateral prefrontal cortex (DLPFC) and orbitofrontal cortex (OFC). A two-tailed p-value of less than 0.05 was considered statistically significant. All analyses were performed using IBM SPSS Statistics, version 24 (IBM Corp., Armonk, NY, USA).

## 3. Results

### 3.1. Correlations between clinical scale scores and emotional perception correction rates

**Fig 3:**
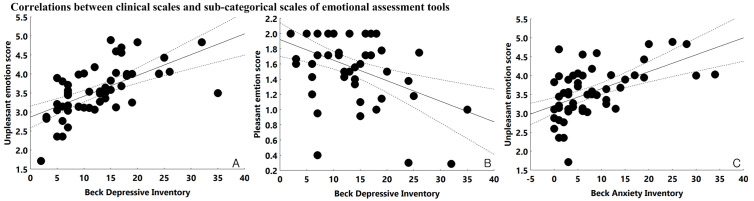
Correlations between clinical scales, emotional perception, and heart rate variability. A: Correlation between Beck Depression Inventory score and correction rate of unpleasant emotional perception, *r* = 0.603, *p* < 0.001. B: Correlation between Beck Depression Inventory score and correction rate of pleasant emotional perception, *r* = –0.438, *p* = 0.001. C: Correlation between Beck Anxiety Inventory score and correction rate of unpleasant emotional perception, *r* = 0.548, *p* < 0.001. D: Correlation between high-frequency power during unpleasant stimulation (unpleasant-HF) and correction rate of unpleasant emotional perception, *r* = –0.417, *p* = 0.002. E: Correlation between standard deviation of NN intervals during unpleasant stimulation (unpleasant-SDNN) and correction rate of unpleasant emotional perception, *r* = –0.406, *p* = 0.002. F: Correlation between root mean square of successive differences during pleasant stimulation (pleasant-RMSSD) and correction rate of unpleasant emotional perception, *r* = –0.423, *p* = 0.001.

Analysis of the clinical scale scores revealed that higher BDI scores were significantly associated with a greater correction rate of unpleasant emotional perception (*r *= 0.537, *p* < 0.001) and a lower correction rate of pleasant emotional perception (*r* = –0.747, *p* < 0.001). A similar pattern was observed with BAI scores, which also demonstrated a significant positive correlation with the correction rate of unpleasant emotional perception (*r* = 0.469, *p* < 0.001) (Fig 3).

### 3.2. Correlations between unpleasant emotional perception and heart rate variability

The correction rate of unpleasant emotional perception was inversely associated with several HRV parameters. Specifically, it showed significant negative correlations with high-frequency power during unpleasant stimulation (unpleasant-HF; *r *= –0.300, *p* = 0.03), unpleasant-SDNN (*r *= –0.389, *p* = 0.003), and pleasant-RMSSD (*r* = –0.377, *p =* 0.004). No significant associations were found between the correction rates of other emotional perception conditions and HRV parameters (Fig 3).

### 3.3. Correlations between emotional perception and prefrontal cortex activation

**Fig 4:**
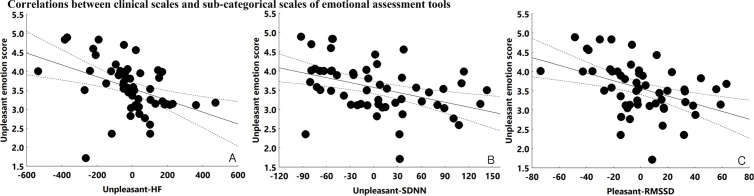
Correlations among emotional perception, heart rate variability, and near-infrared spectroscopy data. A: Correlation between ΔaccHbO₂ in the left DLPFC and correction rate of unpleasant emotional perception, *r* = 0.557, *p* < 0.001. B: Correlation between ΔaccHbO₂ in the left DLPFC and correction rate of pleasant emotional perception, *r* = –0.313, *p* = 0.02. C: Correlation between ΔaccHbO₂ in the right DLPFC and correction rate of pleasant emotional perception, *r* = 0.331, *p* = 0.01. D: Correlation between ΔaccHbO₂ in the left DLPFC and high-frequency power during unpleasant stimulation (unpleasant-HF), *r* = 0.311, *p* = 0.01. E: Correlation between ΔaccHbO₂ in the left DLPFC and standard deviation of NN intervals during unpleasant stimulation (unpleasant-SDNN), *r* = –0.326, *p* = 0.01.

An increased correction rate of unpleasant emotional perception was positively correlated with greater ΔaccHbO₂ in the left dorsolateral prefrontal cortex (*r* = 0.557, *p *< 0.001), suggesting increased cortical engagement in response to negatively perceived stimuli. In contrast, higher correction rates of pleasant emotional perception were associated with decreased ΔaccHbO₂ in both the right (*r *= –0.313, *p *= 0.02) and left dorsolateral prefrontal cortex (*r *= –0.331, *p* = 0.01) (Fig 4).

### 3.4. Correlations between HRV and prefrontal cortex activation during unpleasant emotional stimulation

Finally, HRV parameters during unpleasant stimulation were significantly associated with prefrontal activation. Both unpleasant-SDNN (*r *= –0.326, *p* = 0.01) and unpleasant-HF (*r *= –0.311, *p* = 0.01) were negatively correlated with ΔaccHbO₂ in the left dorsolateral prefrontal cortex, further supporting a link between reduced autonomic flexibility and heightened cortical response to negative emotional stimuli (Fig 4).

## 4. Discussion

### 4.1. Negative emotional perception and clinical scales

The findings indicated that BDI scores were positively correlated with the correction rate of unpleasant emotional perception. Individuals with major depressive disorder (MDD) tend to interpret emotional stimuli more negatively than healthy controls [22–27]. Prior research suggests that individuals with depression often struggle to interpret social cues accurately [28,29] and they are generally more likely to perceive ambiguous stimuli as negative [30]. Mood disorders are known to affect the perception of facial emotional expressions; individuals with such conditions often show reduced sensitivity to positive expressions, such as happiness, while demonstrating a heightened sensitivity to negative expressions, such as sadness [31]. This imbalance may impair their ability to correctly identify mixed emotional expressions [31].

Similarly, BAI scores were also positively associated with the correction rate of unpleasant emotional perception. Anxiety frequently co-occurs with depression [32], and individuals with elevated anxiety levels tend to process negative emotional stimuli more readily [33]. These findings suggest that the emotion perception index developed in this study may serve as a useful proxy for assessing an individual’s mood status.

### 4.2. Unpleasant emotional perception and HRV-HF, SDNN, RMSSD

The analysis revealed that the correction rate of unpleasant emotional perception was negatively correlated with unpleasant-HF, unpleasant-SDNN, and pleasant-RMSSD. According to polyvagal theory [34], the parasympathetic nervous system—regulated via the vagus nerve—plays a critical role in emotional response and adaptive regulation. Therefore, individuals with lower HF, SDNN, and RMSSD values are more likely to experience emotional dysregulation due to insufficient vagal activity. Supporting this, prior studies have demonstrated that healthy individuals typically exhibit decreased HRV during exposure to negative emotional stimuli [35]. Additionally, higher resting HRV has been associated with more effective emotional regulation [36], whereas lower HRV is linked to greater severity of depressive and anxiety symptoms [12,37].

As noted earlier, HRV is strongly associated with mood disorders such as depression and anxiety. In previous work, negative emotional regulation styles in adolescents were significantly correlated with changes in RMSSD reactivity. Furthermore, heart rate itself was found to be negatively associated with negative emotional regulation styles, with adolescents who had more maladaptive regulation strategies displaying both higher and more irregular heart rates [14]. Kemp et al. also reported that individuals with MDD showed significantly lower HRV in time-domain measures and decreased HF power [13]. Other studies have likewise concluded that SDNN, RMSSD, and HF power are markedly reduced in those with depression [38–40].

Although participants in the present study were not clinically diagnosed with MDD, those reporting depressive symptoms demonstrated a tendency toward more negative emotional perception, in line with previous findings. These results underscore the potential utility of HRV as a predictive biomarker for subclinical depression in otherwise healthy adolescents.

### 4.3 Unpleasant emotional perception and DLPFC

The results demonstrated a positive correlation between the correction rate of unpleasant emotional perception and ΔaccHbO₂ within the left dorsolateral prefrontal cortex (DLPFC) (*r* = 0.557, *p* < 0.001). In contrast, the correction rate of pleasant emotional perception was negatively correlated with ΔaccHbO₂ in both the right (*r* = –0.313, *p* = 0.02) and left DLPFC (*r* = –0.331, *p* = 0.01). Groenewold et al. reported that, in individuals with depression, hypoactivation of the left DLPFC was specific to negative stimuli, with no significant changes observed in response to positive stimuli [15]. A meta-analysis by Hamilton et al. further supported the involvement of both the left and right DLPFC in the processing of negative emotions [41]. Other studies have suggested a functional asymmetry, wherein the left prefrontal cortex is more engaged during the experience of positive emotions and the right prefrontal cortex during negative emotions [42]. According to the valence lateralization hypothesis, prefrontal cortical regions are differentially involved in processing approach-related (left hemisphere) versus avoidance-related (right hemisphere) emotional responses [43]. However, contradictory evidence from other studies has shown no significant lateralization in DLPFC activation in response to emotional valence in healthy individuals [44].

Although inconsistencies exist across studies, our findings align with the broader literature indicating differential DLPFC activation patterns depending on emotional valence. While our data showed distinct patterns of activation for negative versus positive emotional perception, these patterns do not significantly diverge from prior research. Further investigation is warranted to elucidate the precise functional contributions of each DLPFC hemisphere to emotional processing.

### 4.4. Heart rate variability and DLPFC

Our results revealed that both unpleasant-SDNN and unpleasant-HF were negatively correlated with ΔaccHbO₂ within the left DLPFC. These findings support growing evidence of an association between prefrontal cortical activity and HRV parameters. As discussed previously, emotion perception and regulation are closely linked to parasympathetic control. Following exposure to emotionally salient stimuli—such as those that evoke negative emotions—the prefrontal cortex may exert top-down regulatory influence via efferent pathways directed toward the amygdala, hypothalamus, brainstem, and medulla [45,46]. Studies utilizing repetitive transcranial magnetic stimulation (rTMS) have shown that stimulation of the DLPFC can reduce blood pressure and attenuate heart rate responses [47]. Specifically, left DLPFC stimulation has been reported to decrease anxiety and promote parasympathetic dominance, reflected by increased RMSSD and reduced LF/HF ratios [45,48].

Collectively, these findings suggest that alterations in HRV are associated with functional changes in the DLPFC, indicating that brain activity elicited by emotional stimuli may be indirectly inferred through autonomic nervous system responses. However, the specific relationship between HRV and localized PFC subregions—particularly the left DLPFC—remains insufficiently understood, highlighting the need for future research to further delineate these mechanisms.

### Limitations

This study has several limitations that should be considered. First, the relatively small sample size limits the generalizability of the findings. Second, the study did not include participants with clinically diagnosed mood disorders, which is essential for evaluating the accuracy and diagnostic validity of the emotion perception test. Future research should aim to include a larger and more diverse cohort comprising both healthy adolescents and individuals with MDD or other psychiatric conditions. In addition, neurotransmitters such as dopamine, serotonin, norepinephrine, gamma-aminobutyric acid, and glutamate are known to play important roles in mood and emotional regulation. Accordingly, future studies should consider incorporating blood-based measurements of neurotransmitter levels to further elucidate the neurobiological underpinnings of emotional perception.

## Conclusions

In summary, the present study found that emotional perception in adolescents—particularly the perception of negative emotions—was significantly associated with individual levels of depression and anxiety. Moreover, negative emotional perception was linked to alterations in both HRV and activity within the left dorsolateral prefrontal cortex (DLPFC). The observed associations between HRV changes and corresponding variations in brain activation further support the interplay between autonomic and cortical mechanisms during emotional processing. These findings suggest that HRV and neuroimaging-derived markers of brain activity may serve as sensitive indicators of emotional perception and its variability in adolescents, particularly in relation to the processing of unpleasant emotional stimuli. As such, these physiological and neural measures may hold promise as objective tools for assessing emotional and mental health risk in this population.
